# Antioxidant Carbocysteine Treatment in Obstructive Sleep Apnea Syndrome: A Randomized Clinical Trial

**DOI:** 10.1371/journal.pone.0148519

**Published:** 2016-02-05

**Authors:** Kang Wu, Xiaofen Su, Guihua Li, Nuofu Zhang

**Affiliations:** Guangzhou Institute of Respiratory Disease, State Key Laboratory of Respiratory Diseases, The 1^st^ Affiliated Hospital of Guangzhou Medical University, Guangzhou, Guangdong, China; Weill Cornell Medical College in Qatar, QATAR

## Abstract

**Objective:**

This study aimed to examine the effects of carbocysteine in OSAS patients.

**Methods:**

A total of 40 patients with moderate to severe obstructive sleep apnea syndrome (OSAS) were randomly divided into two groups. One group was treated with 1500 mg carbocysteine daily, and the other was treated with continuous positive airway pressure (CPAP) at night. Before treatment and after 6 weeks of treatment, all patients underwent polysomnography and completed questionnaires. Treatment compliance was compared between the two groups. Plasma was collected for various biochemical analyses. Endothelial function was assessed with ultrasound in the carbocysteine group.

**Results:**

The proportion of patients who fulfilled the criteria for good compliance was higher in the carbocysteine group (n = 17) than in the CPAP group (n = 11; 100% vs. 64.7%). Compared with baseline values, the carbocysteine group showed significant improvement in their Epworth Sleepiness Scale score (10.18±4.28 vs. 6.82±3.66; P≤0.01), apnea-hypopnea index (55.34±25.03 vs. 47.56±27.32; P≤0.01), time and percentage of 90% oxygen desaturation (12.66 (2.81; 50.01) vs. 8.9 (1.41; 39.71); P≤0.01), and lowest oxygen saturation level (65.88±14.86 vs. 70.41±14.34; P≤0.01). Similar changes were also observed in the CPAP group. The CPAP group also showed a decreased oxygen desaturation index and a significant increase in the mean oxygen saturation after treatment, but these increases were not observed in the carbocysteine group. Snoring volume parameters, such as the power spectral density, were significantly reduced in both groups after the treatments. The plasma malondialdehyde level decreased and the superoxide dismutase and nitric oxide levels increased in both groups. The endothelin-1 level decreased in the CPAP group but did not significantly change in the carbocysteine group. Ultrasonography showed that the intima-media thickness decreased (0.71±0.15 vs. 0.66±0.15; P≤0.05) but that flow-mediated dilation did not significantly change in the carbocysteine group.

**Conclusions:**

Oral carbocysteine slightly improves sleep disorders by attenuating oxidative stress in patients with moderate to severe OSAS. Carbocysteine may have a role in the treatment of OSAS patients with poor compliance with CPAP treatment. However, the efficiency and feasibility of carbocysteine treatment for OSAS needs further evaluation.

**Trial Registration:**

ClinicalTrials.gov NCT02015598

## Introduction

Obstructive sleep apnea syndrome (OSAS) is characterized by repeated episodes of upper airway occlusion during sleep and excessive daytime sleepiness (EDS). It affects at least 4% of adult men and 2% of adult women [1]. OSAS has been identified as having a causal association with cardiovascular disease [2]. Continuous positive airway pressure (CPAP) is currently recognized as the first-line treatment for OSAS. However, poor compliance with CPAP treatment is very common, often because of the cost of CPAP devices and the discomfort associated with their use. Although CPAP can improve symptoms and reduce complications [3, 4], the failure rate of CPAP treatment exceeds 50% [5]. Therefore, there is an urgent need for alternative OSAS treatments.

Because OSAS causes repeated cycles of hypoxia and re-oxygenation that resemble ischemia-reperfusion, the production of reactive oxygen species (ROS) can result from excessive mitochondrial reduction during hypoxia [6]. Several studies have proposed that oxidative stress could underlie the association between OSAS and cardiovascular diseases [7]. Some studies have even reported that OSAS is an oxidative stress disease [8]. Good compliance with CPAP treatment can reduce several cardiovascular risk factors in OSAS patients [4]; however, some studies have shown that CPAP cannot completely repair the oxidant-antioxidant imbalance [9, 10]. Interestingly, antioxidants, such as vitamin C, vitamin E, allopurinol and N-acetylcysteine (NAC), have been shown to positively affect OSAS [11–14]. However, these studies had limitations; for example, most of them enrolled a small number of subjects, and few compared polysomnographic (PSG) parameters, which are important in evaluation of OSAS, before and after treatment. In addition, few reports have compared the outcomes of CPAP use and antioxidant treatments.

Thus, we aimed to identify an antioxidant that is at least as beneficial as previously identified agents for the treatment of OSAS. Carbocysteine could be functionally more effective than some of the other antioxidants currently in use because it scavenges free radicals and replenishes glutathione (GSH), which is a major contributor to antioxidant capacity [15, 16]. In addition, it is less expensive than other antioxidants, including double antioxidant capacity drugs such as NAC. The PEACE study, which was conducted at our institution, found that carbocysteine can be effectively used to treat chronic obstructive pulmonary disease patients. Specifically, carbocysteine reduces exacerbations of the disease and improves quality of life by alleviating oxidative stress and inflammation [17]. Our study was undertaken to evaluate whether the oral antioxidant carbocysteine can effectively reduce oxidative stress and sleep disordered breathing (SDB) in OSAS. This treatment could improve SDB and correct the oxidant-antioxidant imbalance, and it could reduce cardiovascular complications in OSAS patients. Our results indicate that oral carbocysteine has therapeutic potential for patients with moderate to severe OSAS.

## Methods

### 1. Ethics, Study Subjects and Protocol

The study was approved by the Ethics Committee of the First Affiliated Hospital of Guangzhou Medical University (project approval number 2013–19) and was performed according to its guidelines. All of the subjects provided informed written consent. The clinical trial registration number was NCT02015598. Grant support was received from the Science and Technology Planning Project of Guangdong Province, China (2008B03031254). The funders played no role in the study design, data collection and analysis, decision to publish, or preparation of the manuscript.

Subjects were included in the study if they were (1) men aged 18 to 65 years old; (2) diagnosed with obstructive sleep apnea with an apnea-hypopnea index (AHI) ≥15 /h based on the PSG data; (3) non-smokers or had quit smoking ≥6 months prior; and (4) were able to provide consent. Subjects were excluded if they (1) could not tolerate carbocysteine or CPAP; (2) had a history of treatment for OSAS; (3) had an active acute or chronic infection; (4) had been diagnosed with a cardiovascular, neuromuscular, peripheral vascular, or chronic respiratory disease; (5) used steroidal, nonsteroidal anti-inflammatory, or lipid-lowering drugs, vasodilators, cardiovascular medications, or other medications that lower oxidative stress; or (6) used drugs that impair sleep.

**Fig 1:**
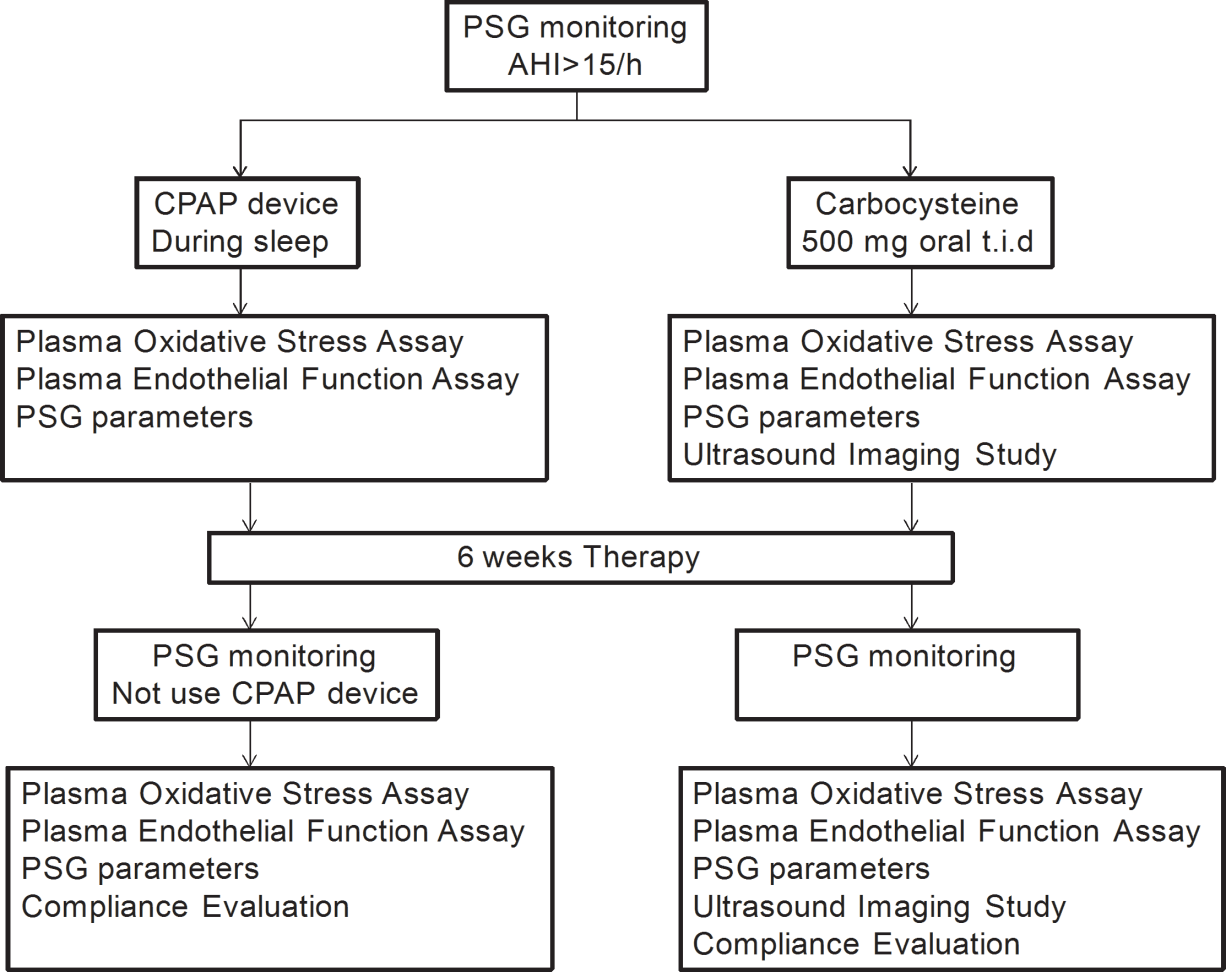
Study plan. PSG **=** Polysomnography; CPAP = continuous positive airway pressure; t.i.d. = three times a day.

Our study enrolled 40 patients with moderate to severe OSAS diagnosed based on overnight PSG studies conducted at a sleep laboratory at the Guangzhou Institute of Respiratory Diseases in Guangzhou, China, from December 2013 to May 2014. The participants were randomly assigned (1:1) to 1 of 2 treatment groups, the carbocysteine group (500 mg oral tablets t.i.d.; Baiyunshan Pharmaceutical, Guangzhou, Guangdong, China) or the CPAP (System One REMstar Auto 557P, Respironics, USA) group, according to simple randomization procedures (using computer-generated random numbers). Blood samples were collected from the participants between 7 and 9 a.m. We also used questionnaires to collect information about patient characteristics, including subjective daytime sleepiness. In addition, the patients in the carbocysteine group underwent endothelial function tests using ultrasonography between 4 and 5 p.m. All of the experimental evaluations were repeated 6 weeks after the CPAP or carbocysteine intervention for all patients. The patients did not use a CPAP device or take oral carbocysteine on the day of PSG monitoring (Fig 1).

### 2. Sleep Studies

The participants were required to complete questionnaires, and their daytime sleepiness was confirmed with the Epworth Sleepiness Scale (ESS)[18] before PSG monitoring began. The PSG monitoring (Alice 5 Diagnostic Device, Respironics, USA) included electro-encephalography (EEG), electro-oculography, electromyography, and electrocardiography and assessments of oronasal flow (thermistor), thoracoabdominal movements, body position, arterial oxygen saturation, and snoring sounds. Skilled staff manually analyzed and scored the electronic raw data according to the standard criteria. Apnea was defined as a significant decrease (>90%) in oronasal flow compared with the baseline for at least 10 seconds. At least 90% of the event’s duration had to meet the amplitude reduction criteria. Hypopnea was defined as an airflow decrease of ≥30% compared with the baseline for at least 10 seconds with ≥4% desaturation from the baseline, or a decrease of ≥50% from the baseline for at least 10 seconds with ≥3% desaturation from the baseline and/or arousal. At least 90% of the event’s duration had to meet the criteria for hypopnea. An oxygen desaturation event was defined as a ≥3% decrease in oxygen. The AHI was defined as the total number of apnea and hypopnea events per hour throughout the total sleep time. According to the American Academy of Sleep Medicine, OSAS severity was based on the AHI and was graded as mild (5≤AHI≤15 /h), moderate (15<AHI≤ 30, n/h), or severe (AHI>30, n/h).

Snoring sounds were monitored simultaneously with the PSG parameters using a piezoelectric snore sensor. The sensor was placed over the suprasternal notch of the trachea to detect the location of the strongest vibrations. The location was marked using adhesive tape. The sound signals were amplified and filtered using a band-pass filter with a frequency range of 70–2000 Hz (to remove the effects of environmental sounds and vascular pulsation noises), a digitized sampling rate frequency of 5000 Hz and a 12-bit A/D converter. All of the snoring episodes were identified by the same analyzer, who rejected other sounds, such as coughing and talking. The analyzer randomly selected 10 stable and regular snoring sound segments for each subject during the full-night PSG study. The mean value of the 10 samples was then used for analysis. A snore contains a range of energy called the power spectral density (PSD). The characteristics of PSD can be used to determine different parameters [19], including the mean (Fmean), median (Fmed), maximum (Fmax), and peak frequencies (Fpeak), with the latter defined as the upper limit of the 95^th^ percentile of the PSD energy frequencies.

### 3. Compliance Evaluation

All of the participants were contacted weekly by telephone to inquire about potential side effects and ensure compliance. Compliance was evaluated at the end of the treatment period. In the carbocysteine group, the medication bottle was retrieved, and the number of remaining pills was counted. Good compliance was defined as a medication usage rate of ≥80%. In the CPAP group, the CPAP device’s memory was downloaded at the end of the study. The CPAP usage and mean AHI data were obtained from data cards inside the CPAP machines. CPAP compliance was defined by the percentage of study days with ≥4 h of CPAP use and an AHI of ≤5/h. The subjects who used the CPAP machine on >70% of the study days were defined as having good compliance.

### 4. Biochemical Analyses

Fasting blood samples were drawn by venipuncture in the morning immediately after PSG and were collected in EDTA-containing tubes. The blood samples were centrifuged and frozen at -80°C until the biochemical analyses.

### 5. Plasma Oxidative Stress Assay

The plasma lipid peroxide levels were estimated using an improved analysis of thiobarbituric acid reactive substance assay, which is primarily performed to measure the malondialdehyde (MDA) level in a sample. Superoxide dismutase (SOD) enzymatic activity was measured using the xanthine oxidase technique. The total GSH concentrations were estimated using an enzyme-labeled immunosorbent assay (ELISA). The biochemical parameters were measured in duplicate, and the mean value was used in analyses. Kits for analyzing MDA, SOD, and GSH were acquired from the Nanjing Jiancheng Bioengineering Institute (Nanjing, China).

### 6. Plasma Endothelial Function Assay

The plasma levels of nitric oxide (NO) compounds (NO_x_; NO_2_^−^; and NO_3_^−^) were measured using the nitric acid deoxidize enzyme method. The endothelin-1 (ET-1) level was measured using ELISA. Kits for analyzing the NO and ET-1 levels were acquired from Nanjing Jiancheng Bioengineering Institute (Nanjing, China).

### 7. Ultrasound Imaging Study

Ultrasound imaging studies were performed using ultrasonography (iU22 xMATRIX Ultrasound System, Philips, USA) for only the carbocysteine group. All measurements were performed at 4 p.m. on the day the PSG study was completed. The patients were examined in a quiet room after a 15-min resting period. A blood pressure cuff was placed 5 cm below the right antecubital fossa. The brachial artery was imaged above the right antecubital fossa in the longitudinal plane using high-resolution ultrasonography to establish the baseline condition. Thereafter, an arterial occlusion was created by inflating a pneumatic blood pressure cuff until the systolic blood pressure was 50 mmHg greater than the baseline pressure for 5 min. This step was followed by rapid deflation of the cuff to increase blood flow through the brachial artery. The brachial artery diameter was measured again 1 min after cuff deflation to define the maximum condition. The absolute change in diameter was defined as the difference in the maximum and baseline diameters. The percentage of flow-mediated dilation (FMD) was calculated as follows: (absolute change/baseline)*100% [20].

The intima-media thickness (IMT) was measured [21] after FMD. Then, the subject rested again for 15 min. With the patient in the supine position, an ultrasound probe was applied longitudinally to the right side of the neck. The measurement was conducted at the far wall of the right carotid artery. Longitudinal scanning was performed from the common carotid artery to the bifurcation of the common carotid artery. IMT was measured within 10 mm proximal to the bifurcation using electronic calipers. Three points were measured in a single scan, and these measurements were synchronized with the R-wave peaks on ECG. Each subject was scanned three times. The mean IMT was calculated based on these nine points. The same experienced investigator, who was blinded to the statuses of the individual patients, performed all of the measurements.

### 8. Outcome Measures

The primary outcome objective was to demonstrate changes in the PSG parameters and oxidative stress biomarkers after 6 weeks of oral carbocysteine intake. The PSG parameters included the ESS score, AHI, T90%, MSaO_2_, LSaO_2_, and snoring parameters. The oxidative stress biomarkers included SOD and MDA.

The secondary outcome objective was to demonstrate the efficacy of oral antioxidant carbocysteine compared with CPAP treatment. An additional secondary endpoint was the change in vascular endothelial function after oral carbocysteine intake. Vascular endothelial function, including FMD and IMT, was measured using high-resolution ultrasonography at baseline and at 6 weeks after carbocysteine treatment.

### 9. Statistical Analysis

The results are presented as the mean±SEM or as the median and 25^th^ and 75^th^ percentiles unless otherwise indicated. The mean differences within and between groups were assessed using the paired and unpaired t-tests, respectively. Differences within and between groups in skewed variables were analyzed using the Mann-Whitney two-sample rank test and Wilcoxon’s signed rank test, respectively. Correlations were assessed with Pearson’s or Spearman’s correlation analysis, as appropriate for normally distributed or skewed variables, respectively. The chi square test was used to compare categorical variables. Fisher's exact test was used if the assumptions of the chi square test were not appropriate. The tests were considered significant at P<0.05. Statistical analyses were performed using Statistical Package for the Social Sciences (SPSS) for Windows (SPSS, version 13.0).

## Results

**Fig 2:**
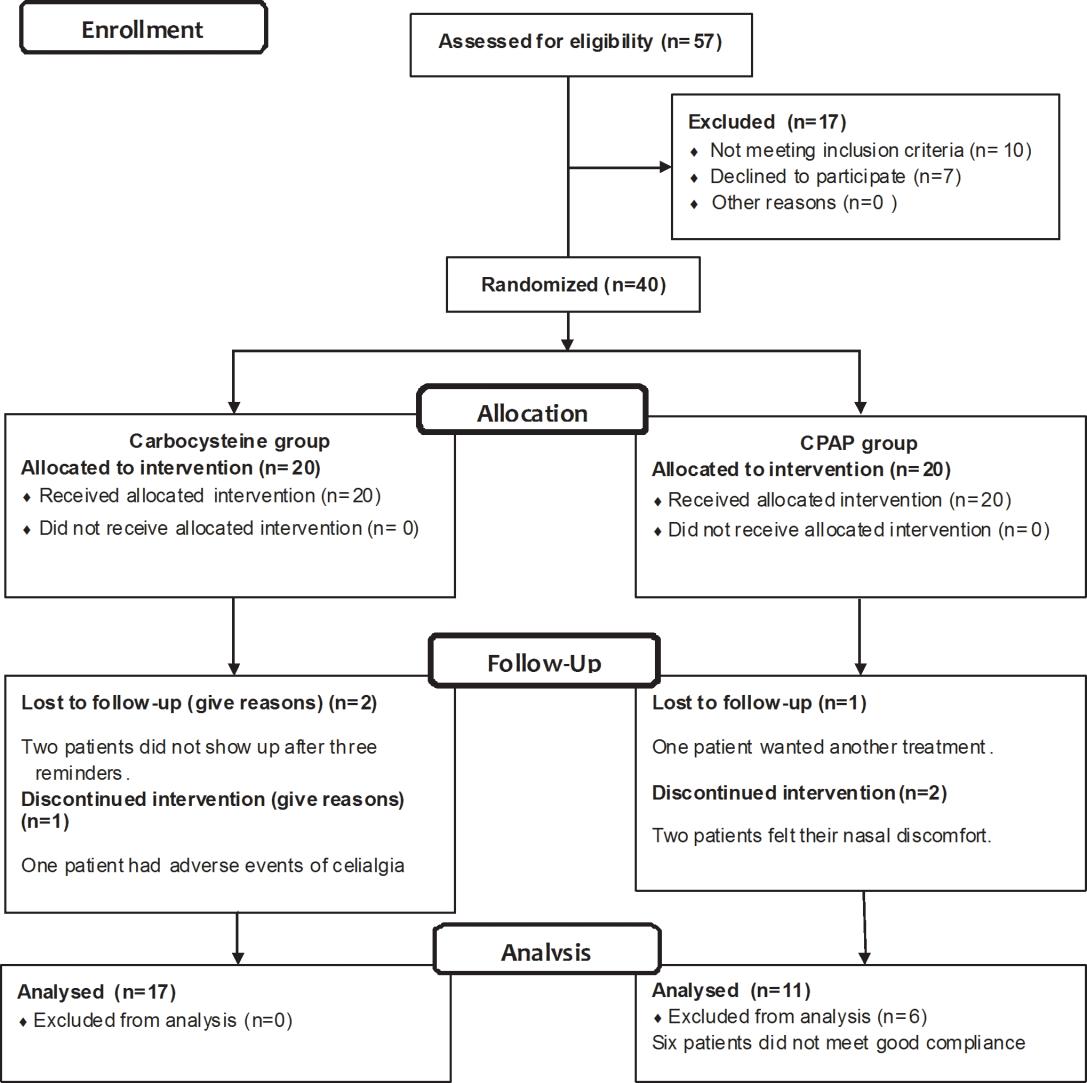
Consort diagram. OSAS = sleep apnea syndrome; CPAP = continuous positive airway pressure.

The 40 patients were randomly divided into two groups of equal size, the carbocysteine group and CPAP group. The characteristics of all subjects are shown in Table 1. Both groups included 3 subjects who were lost to follow-up. In the carbocysteine group, two subjects were withdrawn because they did not show up after three reminders, and one subject was excluded because he reported adverse effects (abdominal pain). In the CPAP group, one patient demanded another treatment, and two patients experienced nasal discomfort while using the CPAP machine. Thus, only 17 patients completed the study in each group (Fig 2).

**Table 1:** Characteristics of the study subjects. ESS = Epworth sleeping scale, AHI = Apnea-hypopnea index, ODI = Oxygen desaturation index, MSaO2 = Mean oxygen saturation, LSaO2 = Lowest oxygen saturation, T90% = Time percentage of 90% oxygen desaturation, Fpeak = Peak frequency. Data are given as means±SEM unless otherwise indicated

Variable	Carbocysteine group (n = 20)	CPAP group(n = 20)	P
**Age**	42.45±12.16	44.45±9.85	0.571
**Neck circumference (cm)**	40.88±2.92	39.68±1.59	0.115
**Waist circumference (cm)**	98.15±10.80	98.60±7.97	0.882
**Body mass index (kg/m**^**2**^**)**	28.31±4.13	27.35±2.16	0.361
**ESS**	10.60±4.11	10.25±3.78	0.781
**AHI(n/h)**	51.82±24.98	50.16±19.25	0.815
**ODI(n/h)**	54.53±26.22	51.88±20.79	0.726
**MSaO**_**2**_**(%)**	94.5 (89;96)	93(92;94.75)	0.346
**LSaO**_**2**_**(%)**	67.45±14.33	69.95±12.19	0.556
**T90%**	10.23(2.48;43.88)	11.47 (5.02;24.68)	0.946

### 1. Compliance Evaluation and Patient Characteristics

The number of remaining pills and the data downloaded from the CPAP device memory indicated that all 17 subjects in the carbocysteine group and 11 subjects in the CPAP group had good compliance. The compliance significantly differed between the two treatments (100% versus 64.6%; P<0.05).

### 2. PSG Parameters

The ESS score, respiratory parameters, and snoring parameters are shown in Table 2. Both CPAP therapy and carbocysteine treatment significantly reduced the ESS score, AHI, T90%, Fpeak, Fmax, Fmed, and Fmean and increased the LSaO_2_ compared with the baseline values (P<0.05, Table 2). However, the oxygen desaturation index (ODI) decreased and the MSaO_2_ increased significantly in the CPAP group (P<0.05, Table 2). This response was not evident in the carbocysteine group (P>0.05, Table 2).

**Table 2:** Polysomnography parameters in patients with OSAS before and after treatment. No significant difference of the baseline and the after treatment value between carbocysteine group and CPAP group.

	Carbocysteine group (n = 17)			CPAP group (n = 11)		
Variable	Baseline	After Carbocysteine	P	Baseline	After CPAP#	P
**ESS**	10.18±4.28	6.82±3.66	≤0.001	11.00±3.32	5.00±3.22	0.003
**AHI (n/h)**	55.34±25.03	47.56±27.32	0.008	54.44±21.52	42.17±19.53	0.002
**ODI (n/h)**	57.95±26.63	53.31±30.06	0.112	56.52±22.87	44.48±21.52	0.003
**MSaO**_**2**_ **(%)**	94(89;96)	95(90,96)	0.143	93(91;95)	94(93,95)	0.027
**LSaO**_**2**_ **(%)**	65.88±14.86	70.41±14.34	0.009	67.36±14.53	73.55±9.70	0.007
**T90%**	12.66 (2.81;50.01)	8.9 (1.41;39.71)	0.004	14.7 (6.31;37.86)	8.76 (3.87;15.99)	0.012
**Fpeak**	70.33±30.48	59.03±32.43	0.005	73.71±21.71	55.71±25.56	≤0.001
**Fmax**	54.53±29.41	42.76±28.09	≤0.001	52.07±23.56	38.98±23.42	0.003
**Fmed**	20.95±12.67	14.78±9.45	≤0.001	19.13±9.60	13.89±8.31	0.009
**Fmean**	16.20±10.13	10.53±6.38	≤0.001	14.41±7.74	10.26±5.80	0.028

# the patients of CPAP group did not use CPAP when monitoring PSG. OSAS = Obstructive sleep apnoea syndrome, CPAP = Continuous positive airway pressure, Fmax = Maximum frequency, Fmed = Median frequency, Fmean = Mean frequency. The Fpeak, Fmax, Fmed and Fmean measures as snoring characteristics of power spectral density energy frequencies. Data are given as means±SEM or median (25th, 75th percentiles)

### 3. Plasma Oxidative Stress Parameters

The MDA levels significantly decreased and the SOD activity levels significantly increased compared with the baseline values in both groups (P<0.05, Table 3). We did not detect a significant difference in the GSH level in either group compared with the baseline level (P>0.05, Table 3).

**Table 3:** Plasma biochemical parameters in patients with OSAS before and after treatment. No significant difference of the baseline and the after treatment value between carbocysteine group and CPAP group.

	Carbocysteine group (n = 17)			CPAP group (n = 11)		
Variable	Baseline	After Carbocysteine	P	Baseline	After CPAP#	P
**MDA (mmol/ml)**	5.42±1.64	4.10±1.52	0.023	5.54±1.07	4.14±0.73	≤0.001
**SOD (U/ml)**	106.24±16.55	118.44±15.10	0.040	108.07±27.68	129.08±18.40	0.015
**GSH (μmol/L)**	38.66±7.93	40.52±43.91	0.437	39.11±6.79	44.36±8.817	0.141
**NO (μmol/L)**	33.74±12.18	40.36±10.08	0.047	33.37±17.42	43.91±15.73	0.012
**ET-1 (pg/ml)**	43.22±3.54	42.27±3.63	0.373	44.17±5.35	40.04±2.67	0.015

# the patients of CPAP group did not use CPAP when monitoring PSG. OSAS = Obstructive sleep apnoea syndrome, CPAP = Continuous positive airway pressure, MDA = Malondialdehyde, SOD = Superoxide dismutase, GSH = Glutathione, NO = Nitric oxide, ET-1 = Endothelin-1.

### 4. Plasma Endothelial Function Parameters

Both the CPAP and carbocysteine treatments significantly increased the NO levels (P<0.05, Table 3). However, the ET-1 level was only significantly decreased in the CPAP group (P<0.05, Table 3), and it showed a non-significant change in the carbocysteine group (P>0.05, Table 3).

### 5. Ultrasound Imaging Study

**Fig 3:**
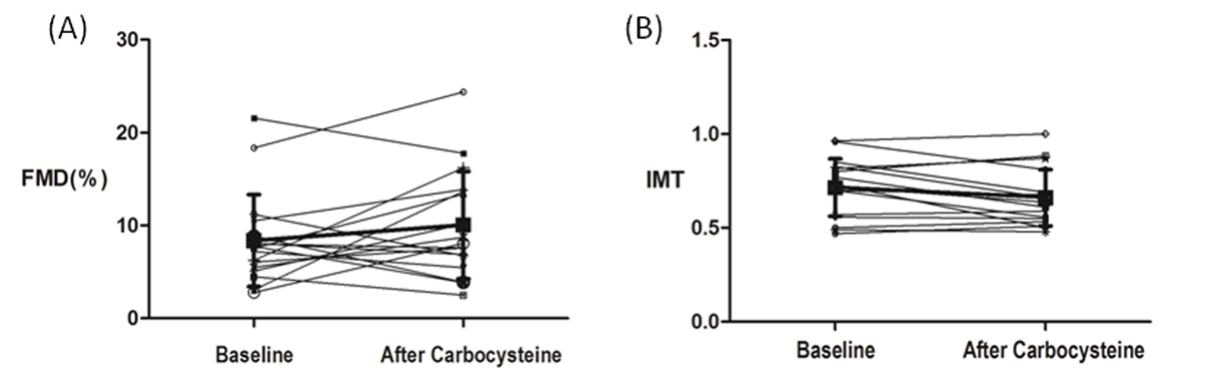
Ultrasound imaging study at baseline and after treatment in the carbocysteine group. (A)Flow-mediated dilation (FMD) of the brachial artery at baseline and after Carbocysteine of 1.5 g daily for 6 weeks in the obstructive sleep apnea group (n = 17; 8.38±4.95 vs. 10.06±5.78; P = 0.182). (B) Intima-media thickness (IMT) of the brachial artery at baseline and after Carbocysteine of 1.5g daily for 6 weeks in the obstructive sleep apnea group (n = 17; 0.71 ± 0.15 vs. 0.66 ± 0.15; P = 0.034)

**Fig 4:**
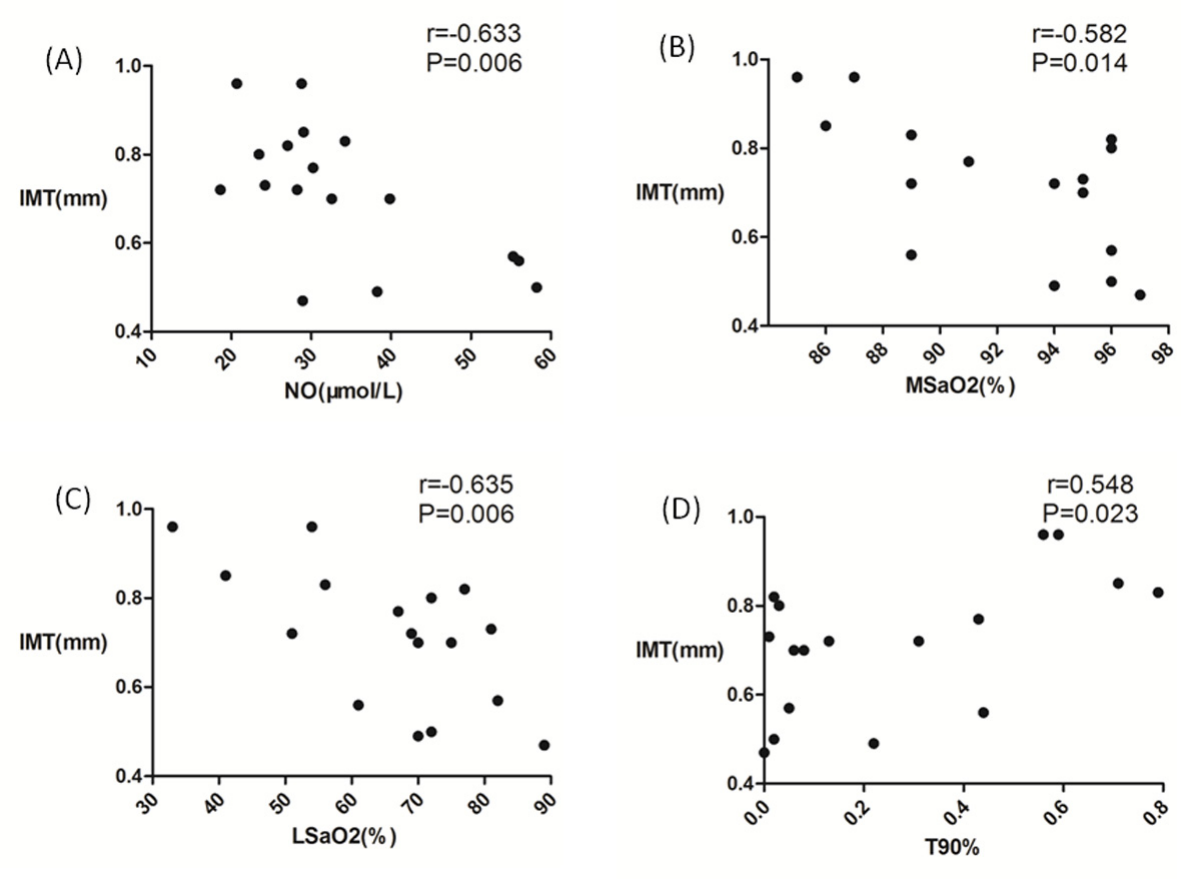
Correlation between intima-media thickness (IMT) and PSG Parameters in OSAS patients at baseline. (A) Pearson correlation analysis between lowest oxygen saturation (LSaO_2_) and baseline IMT. (B) Spearman correlation analysis between Mean oxygen saturation (MSaO2) and baseline IMT. (C) Spearman correlation analysis between time percentage of 90% oxygen desaturation (T90%) and baseline IMT. (D) Spearman correlation analysis between nitric oxide (NO) and baseline IMT.

Carbocysteine therapy significantly affected IMT (P<0.05, Fig 3A). FMD was slightly increased after carbocysteine treatment, but this increase was not significant (P = 0.182, Fig 3B). The baseline IMT was significantly correlated with LSaO_2_ (r = -0.635, P = 0.006), MSaO_2_ (r = -0.582, P = 0.014), T90% (r = -0.548, P = 0.023), and the NO level (r = -0.633, P = 0.006; Fig 4) but not with the AHI, ODI, or SOD activity or the MDA, GSH, or ET-1 levels. FMD was also not correlated with any of the above parameters.

## Discussion

This study showed that OSAS patients who were treated with 1500 mg daily of the oral antioxidant carbocysteine (1) complied better with the treatment than those who were treated with CPAP. Furthermore, (2) carbocysteine significantly improved respiratory parameters and snoring characteristics, (3) reduced oxidative stress, and (4) improved endothelial function.

The upper airways of OSAS patients are narrowed or collapsed, and sleep state-dependent reductions in upper airway dilator motor neuron activity result in repeated episodes of airway occlusion [22]. These recurrent episodes cause short, repetitive cycles of hypoxia-reoxygenation that are associated with increased vascular production of ROS, which promotes oxidative stress. Oxidative stress can affect the tone of upper airway muscles by inhibiting hypoglossal neural output [23], and it can also impair upper airway respiratory muscles [24] and cause soft tissue edema in the upper airway [25]. These conditions can aggravate airway occlusion and create a vicious cycle in OSAS patients. In addition, oxidative stress contributes to impaired endothelial function in these patients [26]. According to previous research, antioxidants can reduce the symptoms of and ameliorate endothelial dysfunction in OSAS. Carbocysteine is a muco-active drug with *in vitro* free radical scavenging and anti-inflammatory properties [16]. It is widely used to treat COPD, pneumonia, rhinitis, and other disorders. However, its use to treat OSAS has not been reported.

Oral carbocysteine is not only convenient, it is also safe and inexpensive. Our study showed that only one of the twenty patients in the carbocysteine group experienced an adverse event (slight abdominal pain). The symptoms abated once this patient stopped taking the medicine. The PEACE study showed that the nature and incidence of adverse events are similar between carbocysteine- and placebo-treated subjects at 1 year [17]. These results indicate that the long-term use of carbocysteine is well tolerated. The commonly used definition of adequate CPAP compliance is ≥4 h of usage per night. This definition is not completely satisfactory because a significant proportion of patients likely require >4 h of usage per night for maximal benefit [27]. OSAS results in respiratory events when patients do not use a CPAP machine. If good compliance had been defined by the overnight use of a CPAP machine, then even fewer patients in the CPAP group would have been considered to have good compliance.

Interestingly, the OSAS patients reported that their snoring was relieved after one or two weeks of treatment with carbocysteine. This finding is similar to those of a previous study, in which NAC was able to decrease snoring, relative snoring time, snoring episodes, and the duration of the longest snoring episodes [14]. However, the volume of snoring that disturbs others has not yet been investigated. Although snore detection is a useful method for monitoring snoring [28], a full-night PSG study can also record snoring volume data using a piezoelectric snore sensor, which is not affected by surrounding noises. This sensor cannot determine the actual number of decibels, but it can be used to semi-quantitatively analyze the data obtained from a self-controlled study. The sound spectrum, which occurs in cycles, might aid in the identification of the acoustics of snoring according to the following properties: (1) the interval and amplitude of snoring are almost identical to and consistent with the breathing rate; (2) the impulse wave of snoring is more regular and periodic than that of the voice; and (3) the short-term energy of snoring is greater than that of the normal voice and is evenly distributed throughout the night [29]. These characteristics could help to discern snoring from other sounds, such as speaking, stridor, and wheezing. In our study, we found that the carbocysteine and CPAP treatments significantly reduced the snoring volume measurements, including the Fpeak, Fmax, Fmed, and Fmean. An increase in snoring volume causes increases in pharyngeal tissue vibration and airflow turbulence, which are relieved with treatment. Snoring is a public health problem that can negatively affect OSAS patients and the people around them, and snoring volume should be considered. However, snore detection is costly and cannot be widely used. Furthermore, the analysis of snoring sounds has not been standardized and lacks guidelines. Viable and simple snoring parameters extracted from PSG data may be used to analyze the changes in self-controlled or semi-quantitative studies. To our knowledge, this study is the first self-controlled study to investigate snoring sounds using only PSG data.

CPAP, the most widely used and effective therapeutic intervention for OSAS patients, improved the PSG parameters and EDS scores in our patient cohort. The antioxidant carbocysteine produced similar effects, except for the changes in the ODI and MSaO_2_. These findings demonstrate that CPAP is superior to oral carbocysteine. However, the carbocysteine group did not show clinically relevant changes in the PSG parameters. In addition, the subjects in the CPAP group did not exhibit clinically significant changes in these parameters when they were studied at night without CPAP; under those circumstances, these patients remained in the severe OSA spectrum. Oxidative stress decreased in both groups after treatment. However, the GSH level did not change after the interventions in either group. Because GSH is an antioxidant enzyme that fights enzymatic oxidation, it may be easily oxidized in OSAS patients.

These results demonstrate that CPAP and carbocysteine might improve symptoms by reducing oxidative stress in OSAS. Upper airway dilation is closely related to OSAS severity. Pharyngeal dilator muscles may weaken or loosen as a result of increased upper airway resistance [22]. We believe that antioxidants can strengthen the tone of the upper airway dilator muscles by enhancing hypoglossal nerve activity. More importantly, antioxidants can reduce soft tissue edema in the upper airway by attenuating oxidative stress in the tissue. Carbocysteine improves the OSAS symptoms caused by both of these factors.

The main mechanism of cardiovascular disease is endothelial dysfunction [30], which is considered a subclinical indicator of vascular or myocardial dysfunction prior to the emergence of clinical signs of overt cardiovascular disease. Previous studies have shown that CPAP treatment results in an increase in NO levels [31]. However, the relationship between OSAS and ET-1, one of the most potent vasoconstriction peptides, is controversial. The plasma ET-1 level is higher in OSAS patients [32]. However, after adjustments for age, sex, oxygen saturation, and cardiovascular disease, the plasma ET-1 level has not been shown to significantly differ between OSAS patients and controls [33]. Our study found that CPAP, but not carbocysteine, leads to a decrease in the ET-1 level. Moreover, CPAP improves endothelial function more efficiently than carbocysteine. We believe that CPAP radically reopens the upper airway, which aids in the recovery of endothelial function by ameliorating respiratory events at night. Although carbocysteine cannot completely reverse the effects of a narrow upper airway, it can effectively reduce oxidative stress, which might prevent cardiovascular complications. And we speculated that CPAP plus antioxidant carbocysteine treatment may be more effective in patients with OSAS.

Ultrasound imaging can provide both structural and functional information about superficial arteries for the direct investigation of endothelial function. FMD and IMT are often assessed in the early detection of vascular disease [34]. Previous investigations have shown that FMD is significantly correlated with atherosclerosis and that this correlation can serve as a non-invasive means of assessing vascular endothelial function [35]. Susie has found that FMD is impaired in OSAS patients compared with controls, even after adjustments for age and BMI [36]. Three months of CPAP treatment has been demonstrated to improve FMD [37]. Other studies have found that antioxidants, such as allopurinol and vitamin C, exerted similar effects [12, 13]. Our study found that carbocysteine improved FMD to a non-significant degree. This lack of significance may have been related to the study’s small sample size. More importantly, a longer treatment time may be required to achieve significant improvements in FMD because changes in the biomarker levels in the body are evident prior to changes in vascular morphology. IMT can be used as an index of systemic atherosclerosis of the carotid arteries, which are the most commonly affected vessels. Ciccone found a positive correlation between IMT and OSAS severity [38]. Reductions in IMT primarily occur during the first 6 months and are sustained for 12 months in patients with reasonable CPAP compliance[39]. Our study revealed that carbocysteine reduced the IMT compared with the baseline value (0.71±0.15 vs. 0.66±0.15 mm). A meta-analysis has shown that a 0.1-mm increase in IMT increases the risk of stroke by 17% and increases the risk of myocardial infarction by 15% [40]. Thus, a 0.05-mm decrease in IMT may reduce the risk of future cardiovascular events after oral carbocysteine treatment. Furthermore, in OSAS patients, baseline IMT has been found to be correlated with nocturnal desaturation but not with the AHI. These results are similar to those of Takahiro’s investigation [41]. We believe that OSAS patients who experience nocturnal desaturation suffer from reduced endothelial function. Additionally, IMT was found to be negatively correlated with the NO level. A reduction in the NO level may result in poor vasomotor dysfunction, increased vascular tension, blood coagulation, and a higher risk of thrombogenesis. All of the above factors highlight the occurrence and development of atherosclerosis, which causes IMT thickening.

In conclusion, the results of our study show that repeated episodes of apnea in OSAS result in oxidative stress. Oxidative stress leads to endothelial dysfunction, which can cause cardio-cerebrovascular complications and exacerbate upper airway obstruction, which creates a vicious cycle. The antioxidant carbocysteine can interrupt this cycle to some extent and improve OSAS severity while reducing cardiovascular complications. Furthermore, oral carbocysteine is safe and inexpensive. However, it cannot fundamentally reverse airway obstruction. In addition, the patients in this study did not show clinically relevant attenuation of the PSG parameters, and they remained within the severe OSAS spectrum after undergoing carbocysteine treatment. These patients continued to suffer from intermittent hypoxia. Thus, carbocysteine should only be used as an alternative treatment for OSAS patients who show poor compliance with CPAP treatment.

### Limitations

Many factors affect snoring characteristics, such as the predominant site of upper airway narrowing, the route of the breath, body position, and sleep stage. However, our study used a self-controlled design, and the random selection of steady snoring episodes could have reduced the effects of these factors. The sample sizes used in this study were relatively small. Further trials involving larger populations are required to confirm our results. Moreover, only males were included, which is an additional limitation. Furthermore, few potential consequences of OSAS, such as decreased endothelial function, were evaluated. Further studies should examine whether several other morbidities that are associated with OSAS and may be effectively treated with CPAP may also benefit from antioxidant treatment. In addition, the benefits of long-term carbocysteine treatment in OSAS need to be assessed. The effects of the combined use of antioxidants and CPAP on OSAS treatment efficacy must also be examined. Furthermore, the OSAS severity level that is the most suitable for antioxidant treatment and the optimum duration of carbocysteine treatment must be determined in future studies.

## Supporting Information

S1 CONSORT Checklist CONSORT Checklist. (DOC)

S1 Protocol Study protocol. (DOCX)
