# Supplementary material for: Antioxidant Carbocysteine Treatment in Obstructive Sleep Apnea Syndrome: A Randomized Clinical Trial
Source: PLoS One. 2016 Feb 5;11(2):e0148519. doi: 10.1371/journal.pone.0148519 (PMC4743936; doi:10.1371/journal.pone.0148519)
Supplement: S1 Protocol — (DOCX) [file pone.0148519.s002.docx]

**1. Abstract**

Obstructive sleep apnoea syndrome (OSAS) is characterized by repeated episodes of upper airway occlusion during sleep. It can cause cycles of hypoxia re-oxygenation. And it was postulated that intermittent hypoxia seems to resemble ischemia-reperfusion which may lead to oxidative stress. It is one of the most important mechanisms of cardiovascular diseases. Some studies have even reported that OSAS is an oxidative stress disease.

Continuous positive airway pressure (CPAP) is currently recognized as the first-line treatment for OSAS. But poor adherence to CPAP treatment is very common. The failure rate with CPAP treatment is more than 50%. So there is an urgent need to find out an alternative treatment for patients with OSAS.

Carbocysteine is an antioxidant. It not only scavenges the free radicals but also replenishes GSH which is the major contributor for the antioxidant capacity. However, Carbocysteine is cheaper than other which has double antioxidant capacity drugs, such as N-acetylcysteine. The purpose is to evaluate efficacy of oral intake of Antioxidant Carbocysteine can reduce oxidative stress and improve the symptom of OSAS. And this treatment can correct the imbalance of oxidant-antioxidant status may reduce cardiovascular abnormalities in patients with OSAS.

**2. Background**

Obstructive sleep apnoea syndrome (OSAS) is characterised by repeated episodes of upper airway occlusion during sleep and excessive day time sleepiness (EDS). It affects at least 4% of adult men and 2% of adult women. However, the prevalence in the range of 30% to 80% in the elderly population.OSAS has been identified as a causal association with cardiovascular disease, including hypertension, coronary artery disease and cerebrovascular accident. What is more, the impact of OSAS can also affect patients’ physical, emotional, intellectual capacities and functional quality of life. Thus, OSAS is a major public health problem due to its high prevalence rate, increased morbidity and mortality, and increased public safety risk. So far, nasal continuous positive airway pressure (CPAP) is recognised the usual first-line of treatment in OSAS. However, poor adherence to CPAP treatment is very common. In one respect is the CPAP device`s cost, and in the other respect is the discomfort associated with its usage. Even though CPAP can improve symptoms, and reduce complications, the failure rate with CPAP treatment is more than 50%. Therefore, it is an urgent need to find out an alternative treatment for patients with OSAS.

Up to now there has been an increasing numbers of evidence linking the association between oxidative stress and endothelial dysfunction in these patients. Since OSAS is characterized by repeated cycles of hypoxia reoxygenation which is seems to resemble ischemia-reperfusion. This process can make the reactive oxygen species (ROS) production increased due to excessive mitochondrial reduction, particularly from activated leukocytes, which occurs during hypoxia . Several studies have proposed that oxidative stress may be an underlying mechanism for the association between OSAS and cardiovascular disease.Even believe that OSAS is an oxidative stress disease.

In a recent investigation, many studies had provided direct evidence that in patients with OSAS, there was a disorder in the oxidant-antioxidant balance due to oxidative stress. And good compliance with CPAP treatment can lower several cardiovascular risk factors in OSA patients. But some studies did not find that CPAP can completely relieve the oxidant-antioxidant balance. So there is more and more studier trying to find the effects of antioxidant in OSAS. Interestingly, vitamin E and vitamin C could not only reduce oxidative stress, but also improve in the SDB and EDS. Moreover, vitamin C and Allopurinol improves endothelial function in OSAS. Furthermore, It is proposed that long-term treatment with N-acetylcysteine (NAC) in patients with OSAS may reduce their dependency pressure on CPAP therapy and sleep disordered breathing (SDB). These preliminary investigations prompted us that antioxidants benefit OSAS. But the studies have some limitations, for instance, most of studies enroll a small number of subjects, and just a few study compared PSG parameters which is important to evaluate OSAS before and after treatment, in addition there is few report about comparing CPAP and antioxidant treatments which can evaluate the effectivity of both difference therapy.

Thus, we try to look for another antioxidant Carbocysteine which is proved to be equally or more beneficial in OSAS and observe its effects on more parameters than the previous investigation compare with CPAP. Carbocysteine may be functionally more effective compared to some of the other antioxidants, as it not only scavenges the free radicals but also replenishes GSH which is the major contributor for the antioxidant capacity. What is more, it is cheaper than other which has double antioxidant capacity drugs, such as NAC. The PEACE study of our institutes has found that Carbocysteine was effective for COPD patients in terms of reductions in exacerbations and improvements in quality of life which is suspected that the effect of alleviating oxidative stress and inflammation. Our study is to evaluate efficacy of oral intake of Antioxidant Carbocysteine can reduce oxidative stress and SDB in OSAS. It recover the imbalance in the oxidant-antioxidant status may reduce cardiovascular abnormalities in patients with OSAS.

**3. Patient selection and consent**

Inclusion criteria: (1) menaged 18 to 65 years old; (2) diagnosed with obstructive sleep apnea with an apnea-hypopnea index (AHI) of ≥ 15 n/h based on the PSG data; (3) non-smokers or had quit smoking ≥6 months prior; and (4) were able to provide consent

Exclusion criteria: (1) could not tolerate carbocysteine or CPAP; (2) had a history of treatment for OSAS; (3) had an active acute or chronic infection; (4) had been diagnosed with a cardiovascular, neuromuscular, peripheral vascular, or chronic respiratory disease; (5) usedsteroidal, nonsteroidal anti-inflammatory, or lipid-lowering drugs, vasodilators, cardiovascular medications, or other medications that lower oxidative stress; or (6) used drugs that impair sleep.

**4. Study Protocol**

The patients were randomly assigned (1:1) into the Carbocysteine (500 mg oral tablets t.i.d.) (Baiyunshan Pharmaceutical, China) or CPAP (System One REMstar Auto 557P, Respironics, USA) group, followed by blood sampling between 7 and 9 a.m. We also used questionnaires to collect the characteristics, including subjective daytime sleepiness. Besides the patients in Carbocysteine group were took endothelial function tests using ultrasonography between 4 and 5 p.m. All the experimental evaluations were repeated 6 weeks after CPAP or Carbocysteine intervention in all the patients. The patients did not use CPAP device or oral Carbocysteine intervention on the day of monitoring PSG.

**5. Methods**

**5.1 Sleep studies**

These patients were required to fill in questionnaire, including name, gender, age, the Epworth sleepiness scale (Epworth sleepiness score, ESS), and medical history, medication history, measuring patients' height, weight, blood pressure, heart rate, blood oxygen, neck circumference, waist circumference, etc, before subjecting them to Polysomnography (PSG)（Alice 5 Diagnostic Device, Respironics, USA). Polysomnographic studies included recording of electro-encephalogram (EEG), electro-oculogram (EOG), electromyogram (EMG), oronasal flow, thoracoabdominal movements, electrocardiogram(ECG), body position, arterial oxygen saturation and snoring sounds. Page-by-page analysis and scoring of the electronic raw data were performed manually. The datum according to the standard criteria were analysed manually by skilled staff。 Apnoea was defined as a significant decrease (> 90%) in oronasal flow of baseline for at least 10 seconds. At least 90% of the event’s duration had to meet the amplitude reduction criteria. Obstructive apnea event associated with inspiratory effort throughout the whole period of absent airflow. Hypopnoea was defined as a airflow drop≥30% of baseline for at least 10 seconds with ≥ 4% desaturation from prevent baseline or a drop ≥ 50% of baseline for at least 10 seconds with ≥3% desaturation from baseline or and arousal. At least 90% of the duration of events met the criteria for hypopnoea. Oxygen desaturation events were defined as drop in oxygen ≥ 3%. AHI was defined as the sum number of apneas and hypopneas per hour in total sleep time. The severity of OSAS was graded based on the AHI as mild (5≤AHI≤15, n/ h), moderate (15<AHI≤30, n/ h), and severe (AHI>30, n/ h). The oxygen desaturation index (ODI) was defined as the number of desaturation events per hour of sleep. Time percentage of 90％oxygen desaturation (T90%) was defined as the time percentage of 90％oxygen desaturation during sleep.

The snoring sounds were collected simultaneously with PSG by a piezoelectric snore sensor. The sensor was placed over the suprasternal notch of trachea to detect the strongest vibration part of the sounds, and then was fixed by adhesive tape. The sounds signal were amplified and filtered using a band-pass filter with the frequency range of 10–100 Hz (to remove the effect of environmental sounds and vascular pulsation noises) and digitized sampling rate frequency of 5000 Hz and a 12 bit A/D converter. The snoring episodes were identified by the same analyzer to reject other sounds, such as cough, voice and so on. The analyzer randomly selected 10 times of the snores which were stable and regular in each subject during full-night PSG study. And then the mean value of the 10 times samples was analyzed. The Alice5 software can collect power energy in every 0.002 seconds of each snore. And a snore contains a series of energy which is called power spectral density (PSD). The characteristic of the PSD can be set different parameters: the mean (Fmean) , median(Fmed), maximum (Fmax) and peak (Fpeak, the last defined as the upper limit of the 95th percentile of PSD energy) frequencies.

**5.2 Biochemical Analyses**

Fasting blood samples were drawn by venipuncture in the morning immediately after PSG into EDTA-containing tubes. Blood samples were immediately centrifuged (3000rmb/min) and frozen at -80°C until biochemical assay.

**5.2.1 Plasma Oxidative Stress Assay**

Lipid peroxides levels in the plasma were estimated with an improved analysis of TBARSs, which primarily reflects malondialdehyde (MDA). Superoxide dismutase (SOD) enzymatic activity was measured by xanthine oxidase technique.Total glutathione (GSH) concentration was estimated by the method of enzyme-labelled immunosorbent assay .The biochemical parameters were measured in duplicate and the mean value used in the analyses. The kits for measuremnt of MDA, SOD, and GSH were from Nanjing Jiancheng Bioengineering Institute (Nanjing, China).

**5.2.2 Plasma Endothelial Function Assay**

Plasma levels of nitric oxide(NO) compounds (NOx; NO2−+ NO3−) were measured with nitric acid deoxidize enzyme method . Endothelin-1(ET-1) was measured with a commercially available ELISA kit. The kits for measuremnt of NO, ET-1 were from Nanjing Jiancheng Bioengineering Institute (Nanjing, China).

**5.3 Ultrasound Imaging Study**

Ultrasound Imaging Studies were measured by ultrasonography(iU22 xMATRIX ultrasound system ,Philips,USA) only in Carbocysteine group. All measurements were performed at 4:00 p.m. on the day of completion of the sleep study. The patients were investigated in a quiet room. after a resting period of 15 min. A blood pressure cuff was placed 5 cm below the right antecubital fossa. Vessel diameter was measured by high-resolution ultrasonography of the brachial artery at the right arm. The vessel diameter was measured under resting baseline conditions. Thereafter, arterial occlusion was created by inflation of the pneumatic blood pressure cuff to a pressure of 50 mmHg above baseline systolic blood pressure for 5 min. This was followed by rapid deflation of the cuff to induce a brief high flow state through the brachial artery. Repeat measurement of brachial artery diameter was made 1 min after cuff deflation as maximum condition. Data were reported as baseline diameter, absolute change in diameter (maximum minus baseline) and percentage change in diameter [(absolute change/baseline)*100%], known also as percentage flow-mediated vasodilation (FMD)

IMT was measured from a B-mode, high-resolution ultrasound images on a real-time basis after FMD. The subject had another resting period of 15 min. With the subject in the supine position, an ultrasound probe was applied longitudinally to the surface of the skin on the right side of the neck. The measurement was applied to the far wall of the right carotid artery. Longitudinal scanning was performed from the common carotid artery to the bifurcation of the common carotid artery. Scanning was performed in the optimal position. An ECG monitor integrated with the ultrasound machine was also applied. The ultrasound images were recorded on the videotape. After the bifurcation of the common carotid artery was confirmed, IMT was measured from the B-mode scan with electronic calipers to within 10 mm proximal to the bifurcation. Three points were measured in one scan, which was synchronized with the R-wave peaks on the ECG to avoid possible errors resulting from variable arterial compliance. Three scans were performed for each study subject. Mean IMT was calculated from night points. All measurements were performed by the same experienced investigator, who was blinded to the status of the individual patient.

**5.4 Adherence Evaluation**

A weekly telephone call was made to all participants in order to enquire about potential side-effects and insure the adherence. At the end-of-treatment visit, the adherence was evaluated. At the Carbocysteine group, the medication bottle was retrieved and the number of remaining pills counted. The good adherence was defined the usage rate of medication ≥80%. At the CAPA group, the CPAP device memory was downloaded at the end of the study. The data of CPAP usage and mean AHI were obtained from data cards inside the CPAP machine. Compliance with CPAP was defined as percent of days with CPAP usage for ≥ 4 hours among the total days of the study. Subjects who showed good compliance (>70% of days) with the CPAP device were defined as good adherence.

**The place of the study runs from**

Guangzhou institute of respiratory diseases, Guangzhou, China.

**The date range for the study**

The study is expected to run from December 2013 to May 2014.
